# Experimental Demonstration of the Fitness Consequences of an Introduced Parasite of Darwin's Finches

**DOI:** 10.1371/journal.pone.0019706

**Published:** 2011-05-11

**Authors:** Jennifer A. H. Koop, Sarah K. Huber, Sean M. Laverty, Dale H. Clayton

**Affiliations:** 1 Biology Department, University of Utah, Salt Lake City, Utah, United States of America; 2 Mathematics Department, University of Utah, Salt Lake City, Utah, United States of America; University of Liverpool, United Kingdom

## Abstract

**Background:**

Introduced parasites are a particular threat to small populations of hosts living on islands because extinction can occur before hosts have a chance to evolve effective defenses. An experimental approach in which parasite abundance is manipulated in the field can be the most informative means of assessing a parasite's impact on the host. The parasitic fly *Philornis downsi*, recently introduced to the Galápagos Islands, feeds on nestling Darwin's finches and other land birds. Several correlational studies, and one experimental study of mixed species over several years, reported that the flies reduce host fitness. Here we report the results of a larger scale experimental study of a single species at a single site over a single breeding season.

**Methodology/Principal Findings:**

We manipulated the abundance of flies in the nests of medium ground finches *(Geospiza fortis*) and quantified the impact of the parasites on nestling growth and fledging success. We used nylon nest liners to reduce the number of parasites in 24 nests, leaving another 24 nests as controls. A significant reduction in mean parasite abundance led to a significant increase in the number of nests that successfully fledged young. Nestlings in parasite-reduced nests also tended to be larger prior to fledging.

**Conclusions/Significance:**

Our results confirm that *P. downsi* has significant negative effects on the fitness of medium ground finches, and they may pose a serious threat to other species of Darwin's finches. These data can help in the design of management plans for controlling *P. downsi* in Darwin's finch breeding populations.

## Introduction

Introduced parasites and pathogens are an increasing problem as economic growth and trade provide further opportunities for species to invade [1]. Small, endemic populations of hosts, such as those on islands, are particularly at risk from introduced parasites and pathogens because extinction can occur before hosts have a chance to evolve effective defenses [2], [3]. For example, the introductions of avian malaria and its mosquito vector to the Hawaiian Islands have been implicated in the rapid extinction of several endemic honeycreeper species [4], [5], [6]. The Galápagos Islands have fared better; none of the birds endemic to this archipelago have suffered extinction due to parasites or pathogens over recorded history [7]. However, recent pressure from introduced parasites and pathogens has the potential to cause serious population declines, if not extinctions [8], [9].

A parasite of particular concern is the recently introduced fly, *Philornis downsi* (Diptera: Muscidae; Dodge & Aitken) [10]. To our knowledge, there are no studies of the fitness consequences of *P. downsi* on hosts within the native range of this fly. Aside from the Galapagos, the only other records of *P. downsi* are from Trinidad and Brazil [11]. *P. downsi* was not observed in the nests of birds in the Galapagos until 1997 [12]. *P. downsi* is now known to parasitize at least 14 species of Galápagos land birds, including 9 species of Darwin's finches [12], [13], [14]. It has been found on 11 of the 13 Galápagos Islands sampled [15]. *P. downsi* may be partly responsible for recent declines of the endangered mangrove finch (*Camarhynchus heliobates*), the endangered medium tree finch (*Camarhynchus pauper*), and the warbler finch (*Certhidea fusca*) [8], [9], [13].

**Figure 1:**
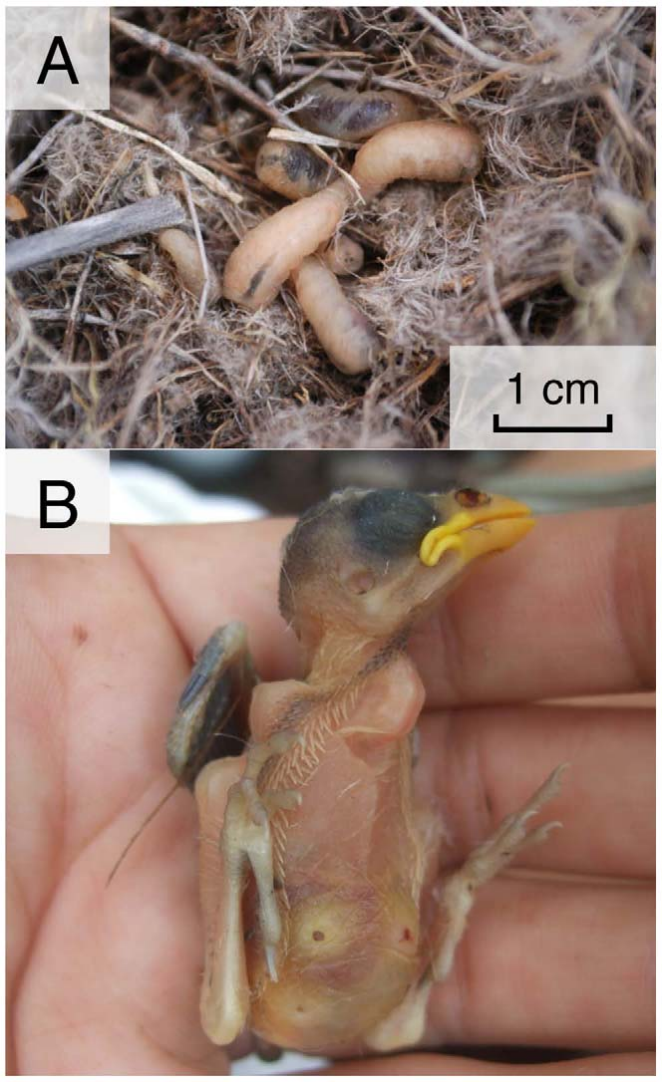
Study organisms. A) *Philornis downsi* larvae in the nest of a medium ground finch *(Geospiza fortis)*; photo courtesy of A. Hendry; B) *G. fortis* nestling with three lesions on the abdomen and damage to the nares (nostrils) from *P. downsi* larvae.

*P. downsi* is an obligate nest parasite of birds. While the adult flies are non-parasitic (they feed on decaying matter), the larvae are semi-hematophagous parasites of nestlings [16] (Fig. 1A). *P. downsi* larvae chew through the skin of nestlings and consume blood and other fluids [16] (Fig. 1B). Larvae feed primarily at night; during the day most larvae burrow into the nest material [17]. Adult flies lay their eggs in the nesting material and nares (nostrils) of nestlings [18], [19]. After the eggs hatch, the larvae complete three instars, the first of which can live in the nares of the host or freely in the nest material. Damage to the nares of nestlings can persist into adulthood [20]. Second and third instar larvae live freely in the nest material, where they eventually pupate and later emerge as adult flies.

Earlier studies of the impact of *P. downsi* on Darwin's finches identified this parasite as a potential threat (Table 1). Several studies report a negative correlation between *P. downsi* abundance and fledging success [21], [22], [23], [24]. Additional studies report varying degrees of nest failure (complete or partial brood loss) based on finding *P. downsi* in nests [12], [13], [14], [19]. While these studies have been integral in bringing attention to the impact of *P. downsi* on various finch species, the next step is to measure the direct effect of the parasite, while controlling for other variables that may be contributing to nest failure (e.g. ecological variables such as rainfall and food availability, which differ from year to year [25], [26]).

**Table 1:** Tests of the impact of *Philornis downsi* on Darwin's Finches.

Darwin's Finch Species	Nestling Hb Level	Nestling Growth	Fledging Success			Reference
			Obs*	Cor†	Exp‡	
*Geospiza fortis*	-	N	-	Y	-	[23]
*Geospiza fuliginosa* α	Y	-	-	Y	-	[21]
*Geospiza fuliginosa*	-	-	-	Y	-	[24]
*G. fortis & fuliginosa* β	Y	Y	-	-	Y	[29]
*G. fortis, fuliginosa & scandens* β	-	-	Y	-	-	[19]
*Camarhynchus pauper*	-	-	Y	-	-	[13]
*Camarhynchus heliobates*	-	-	Y	-	-	[14]
4 species (3 genera)β ^,^ Ω	-	-	Y	-	-	[12]
6 species (4 genera)β ^,^ θ	-	-	-	Y	-	[22]

(Y, impact of parasite on host parameter detected; N, no impact detected; -, not tested).

*Observational data suggest *P. downsi* responsible for nestling mortality.

† Correlational data show a negative relationship between parasite abundance and fledging success.

‡ Experimental nests fumigated to reduce parasite abundance.

α Different islands pooled for analysis.

β Different species pooled for analysis.

Ω Geospiza fuliginosa, Camarhynchus parvulus, Cam. psittacula, Certhidea olivacea.

θ Geospiza fuliginosa, G. fortis, Camarhynchus parvulus, Cam. psittacula, Cactospiza pallida, Certhidea olivacea.

To measure the magnitude of a parasite's direct effect on a host, an experimental approach is necessary [27], [28]. Correlations between parasite abundance and host fitness can be difficult to interpret because they do not measure the direct effect on host fitness. For example, poorly fed birds can have high numbers of parasites because they have little energy to invest in defense, while also having low reproductive success because they have little energy to invest in offspring. The consequence is a spurious correlation (or at least an inflated one) between parasite abundance and host fitness.

To date, just one published study has experimentally manipulated *P. downsi* abundance and measured its impact on Darwin's finches. Fessl *et al.* [29] eliminated *P. downsi* from four *Geospiza fortis* nests, and eight *G. fuliginosa* nests, by fumigating the nests with a 1% pyrethrin solution. Following treatment, the authors monitored nestling growth over a four-day period; they also monitored nestling hemoglobin level and the fledging success of each nest, compared to non-fumigated nests. Though limited sample sizes required them to pool data between species and across years, their results showed that nestlings in fumigated nests tended to have higher hemoglobin concentrations, a significantly higher growth rate, and significantly greater fledging success than nestlings in non-fumigated nests (Table 1).

Here we report the results of a larger scale experimental study of a single species of Darwin's finch at a single site over a single breeding season. We manipulated the abundance of flies in the nests of medium ground finches *(Geospiza fortis*) and quantified the impact of the parasites on nestling growth and fledging success.

## Materials and Methods

### Ethics statement

All procedures were approved by the University of Utah Institutional Animal Care and Use Committee (protocol #07-08004).

### Study site and experimental design

Our study was conducted January-April, 2008 at El Garrapatero on Santa Cruz Island in the Galápagos Archipelago, Ecuador. *G. fortis* is abundant at this site [23], where it builds nests in endemic tree cacti (*Opuntia echios gigantea*) and *Acacia* trees, 1.5 to 4 meters above the ground. Clutch size ranges from 2–5 eggs. The incubation period is approximately 12 days, and nestlings spend 10–14 days in the nest prior to fledging. Both sexes of *G. fortis* feed nestlings and clean the nest, but only females incubate eggs and brood hatched offspring. Breeding pairs of adults often re-nest, but they do not use the same nest again [26].

We searched a 1.5 km×1.5 km area for active *G. fortis* nests throughout the breeding season. We monitored a total of 48 nests, all of them constructed in tree cacti, by 34 different breeding pairs of finches. Fourteen (29%) of the nests in our sample were repeat bouts of nesting during the study period. Adult birds were netted near the nest and fitted with a numbered Monel metal band and three plastic color bands for identification at a distance. Active nests were visited every other day between the hours of 0600 and 1100, and the number of eggs and nestlings were recorded. Nests were included in the experiment if they were discovered before the eggs hatched (n = 44 nests) or, in the case of four nests, soon after hatching (nestlings ≤5 days of age, but these four nests were omitted from all analyses of growth). We continued to check nests and process nestlings (see below) until the oldest nestling was 10 days of age, or until all of the nestlings died. Processing nestlings older than 10 days of age can trigger premature fledging [30]. Therefore, once the oldest nestling reached 10 days of age, we stop processing nestlings. *G. fortis* nests have a side entrance that makes it possible to census older nestlings from a distance with binoculars. Once empty, nests were collected to count parasites.

Nests were randomly assigned to the experimental group (n = 24 nests) or control group (n = 24 nests). In most cases of re-nesting by a single pair of birds, the treatment was reversed between reproductive bouts. The floors of experimental nests were fitted with a liner constructed from a small section of nylon stocking stretched over a wire hoop (∼9 cm in diameter). The liner prevented most of the fly larvae in the bottom of the nest from reaching the nestlings. This approach has been effective in other experimental manipulations of nest parasites [31]. Experimental nests were fitted with liners within one day of the first egg hatching (a clutch of eggs normally hatches over two to four days). The four nests that already contained nestlings when first monitored were all assigned to the unlined group because they could have already been exposed to parasites. Parasite larvae occasionally crawled over the liners, coming into contact with nestlings. For this reason, liners were carefully examined and cleaned or replaced each time the nests were checked. Any larvae found and removed were included in final counts of parasite abundance, since these parasites may have been able to feed on nestlings and may have affected nestling growth and survival.

### Nestling growth

At each nest check the nestlings were weighed with a digital balance (Ohaus, 0.1 g accuracy). In addition, the following measurements were taken with digital calipers (Fisherbrand, 0.01 mm accuracy): bill length, bill depth, bill width, tarsus length, and length of the outermost primary feather from where it emerged from the skin to its distal tip. At the first visit after hatching, nestlings were aged based on body mass using data from Boag [32], as follows: ≤1.9 grams (1 day old); 2–2.9 grams (2 days old); 3–3.9 grams (3 days old). New nestlings were marked individually by coloring a toenail with a permanent marker. At three to four days of age they were given a single plastic color band. When nestlings were at least seven days of age they were fitted with a numbered Monel metal band and three plastic color bands.

Because Darwin's finches have asynchronous hatching, the fact that we processed nests on alternate days meant some birds (“odd day birds”) were processed for the first time at one day of age - and on odd days thereafter - until they were nine days old. Other birds (“even day birds”) were processed for the first time at two days of age - and on even days thereafter - until they were ten days old. These two data sets were used to construct growth curves for lined and unlined treatments.

### Fledging success

Fledging was confirmed by observing and identifying birds on the basis of their color bands after they left the nest.

### Parasite abundance

After each nesting bout we removed the nest and placed it in a sealed plastic bag. The nest was carefully dissected within eight hours of collection and *P. downsi* larvae, pupae, and eclosed pupal cases were counted. First instar larvae, which are too small to discern reliably in the nest material, were not included in counts of parasite abundance. Total parasite abundance was the sum of second and third instar larvae, pupae, and eclosed pupal cases. Other types of fly larvae, e.g. Sarcophagidae, were identified but not included in counts of total parasite abundance because these larvae are not parasitic; they feed on the tissues of dead nestlings [29].

### Statistical analyses

Statistical analyses were done in Prism® v.5.0b (GraphPad Software, Inc.) and R v.2.12.2 (R Development Core Team). Nestling growth was analyzed using regressions and two-tailed t-tests. For some growth parameters we also calculated effect size, i.e. the mean difference in a growth parameter between the lined and unlined treatments [33]. We used bootstrapping (10,000 repetitions) to construct 95% confidence intervals around mean effect sizes [33].

It was not possible to analyze growth over time using repeated measures ANOVA or GLMM because extensive mortality in one of the groups (>80% prior to fledging in unlined, heavily parasitized nests) made sample sizes very uneven over time. Therefore, growth data were tested for an effect of treatment simply by comparing the final values taken for lined nests and unlined nests, when nestlings were nine or ten days old. Thirteen nestlings in seven unlined nests survived to at least nine days of age compared to 26 nestlings in twelve lined nests. To avoid pseudoreplication, we used the mean brood value of nine and ten day old nestlings in each nest. The data for nine and ten day old birds were combined for analysis unless there was an effect of age on the growth parameter of interest (determined via regression analysis). There was an effect of age only in the case of outermost primary feather length, which still had not begun to asymptote by Days 9 and 10 (R^2^ = 0.30, p = 0.003). Therefore, the feather data were analyzed separately for nests containing nine and ten day old nestlings.

## Results

### Parasite abundance

**Figure 2:**
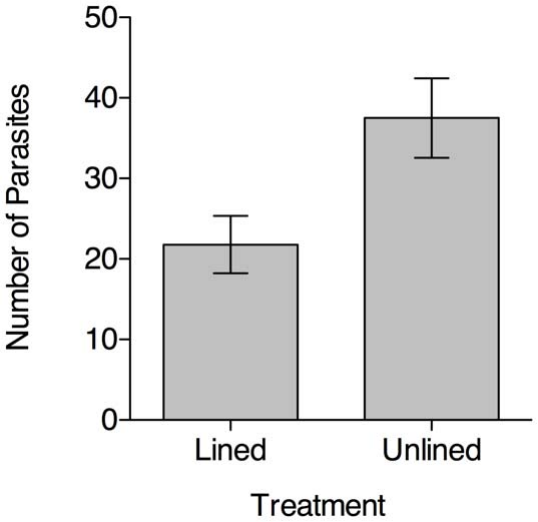
Comparison of the mean (±SE) number of *P. downsi* in lined and unlined nests.

*P. downsi* was present in 43 of 48 *G. fortis* nests (90%). Liners presumably did not prevent adult flies from laying eggs in nests; however, if liners reduced the number of opportunities for larvae to feed, then lined nests should have had fewer parasites than unlined nests. In support of this prediction, we found that lined nests had significantly fewer parasites per nest than unlined nests (mean parasite load ± SE = 21.79±3.56 in lined nests, compared to 37.50±4.92 in unlined nests; Welch's t-test, t = 2.58, df = 41, p = 0.01 (Fig. 2)).

### Nestling growth

**Figure 3:**
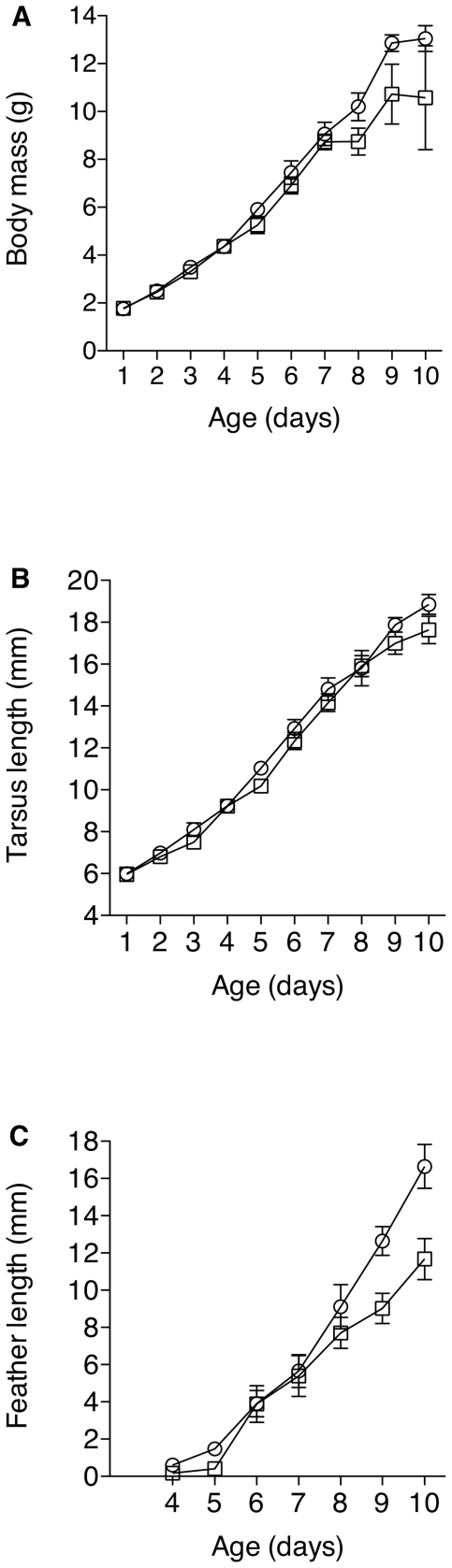
Comparison of mean (±SE) growth parameters for nestlings in lined (◯) and unlined (□) nests, including body mass (A), tarsus length (B), and outermost primary feather length (C).

Nestlings in lined nests were not significantly heavier than nestlings in unlined nests (t = 1.73, df = 18, p = 0.10; Fig. 3A). However, an analysis of effect size revealed that nestlings in lined nests (mean ± SE, 12.7±0.4 g) were 1.7 g heavier, on average, than nestlings in unlined nests (11.0±1.0 g), with a 95% CI = −0.3 g to 3.7 g. Thus, nestlings in lined nests could range from 3.7 g heavier than nestlings in unlined nests, to 0.3 g lighter; however, they were lighter in only 5% of the bootstrap samples.

Tarsus length did not differ significantly between nestlings in lined (18.14±0.34 mm) versus unlined nests (17.23±0.45 mm) (t = 1.64, df = 18, p = 0.12; Fig. 3B). However, analysis of effect size showed that nestlings in lined nests had tarsi 0.91 mm longer than nestlings in unlined nests (95% confidence interval = −0.09 mm to 1.97 mm). The 95% CI around this effect size indicated that nestlings in lined nests could have tarsi up to 1.97 mm longer, on average, than nestlings in unlined nests. Alternatively, nestlings in lined nests could have tarsi up to 0.09 mm shorter than nestlings in unlined nests, but only in 4% of the bootstrap samples.

Outermost primary feathers of “odd day” nestlings in lined nests (12.64±0.77 mm) were significantly longer than those of nestlings in unlined nests (9.02±0.82 mm) (t = 3.13, df = 13, p = 0.008; Fig. 3C). Outermost primary feathers of “even day” nestlings in lined nests (16.65±1.18 mm) were also significantly longer than those of nestlings in unlined nests (11.67±1.10 mm) (t = 2.27, df = 10, p = 0.05).

A composite measure of bill size, using a principal components analysis of bill length, bill width, and bill depth [26], revealed that PC1 explained 68.5% of the variation (eigenvalue = 2.05). However, PC1 did not differ significantly between nestlings in lined and unlined nests (t = 0.831, df = 18, p = 0.42), nor was there a strong trend.

### Fledging success

**Figure 4:**
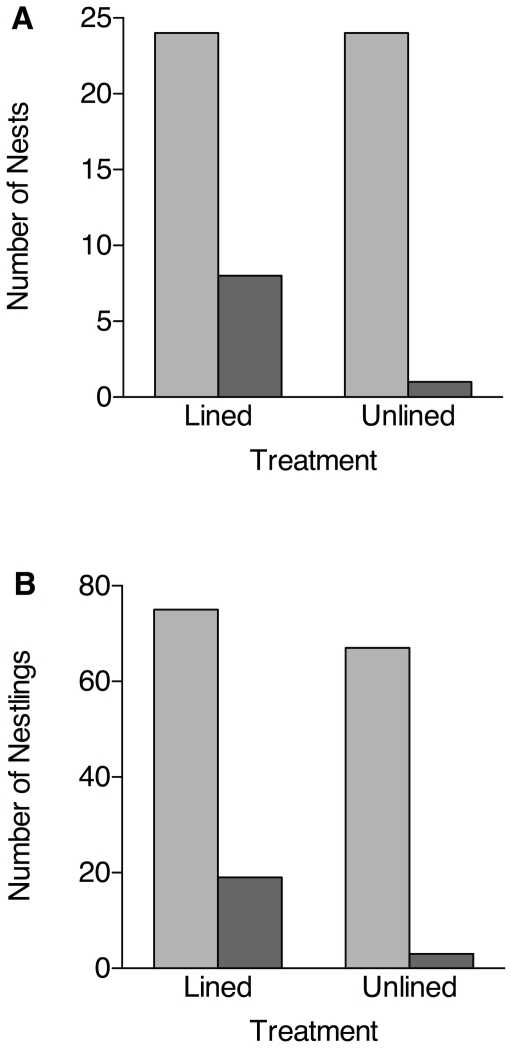
Effect of liners on host fledging success. Light bars are the total number of (A) nests and (B) nestlings monitored. Darker bars are (A) the number of nests that fledged one or more young, and (B) the total number of fledglings from nests in each treatment.

Nestlings in lined nests had significantly greater fledging success than nestlings in unlined nests. Eight of 24 lined nests (33%) fledged young, compared to just one of 24 (4%) unlined nests (Fisher's exact test, p = 0.02, Fig. 4A). We also compared the number of individual nestlings that fledged from lined versus unlined nests: 19 of 75 nestlings (25%) from lined nests successfully fledged, compared to only three of 67 nestlings (4%) from unlined nests (p<0.001; Fig. 4B). Thus, the experimental reduction in parasite number had a clear positive impact on fledging success.

## Discussion

Our study is a rigorous experimental test of the impact of *P. downsi* on the fitness of Darwin's finches. Our experimental design minimized variation between species, sites and years, allowing us to quantify the direct effect of *P. downsi* on parameters of host fitness. We manipulated parasite abundance in a relatively large number of medium ground finch nests using nest liners, rather than chemical fumigants, thus eliminating any possible side effects of pesticides on nestling growth or other fitness components [34]. Liners reduced parasite abundance by 42%, on average. This reduction in parasite load led to a significant increase in the number of nests that successfully fledged young. Our results are consistent with those of Fessl *et al.* [29], who also found a significant increase in the number of nests that successfully fledged young when parasites were completely eliminated through the use of a fumigant.

Our study further suggests that *P. downsi* has a negative effect on nestling growth. When we tested the impact of experimental treatment on nestling size using outermost primary feather length as an index of growth, there was a clear difference. Nestlings in unlined nests had outermost primary feathers that were 30% shorter than nestlings in lined nests, indicating that birds fledging from unlined nests would have underdeveloped feathers. Feather length is a sensitive measure of growth in birds, because feathers grow more rapidly than overall body mass or tarsus length [32], [35], [36].

Nestlings in unlined nests also tended to have lower body mass, and shorter tarsi, than nestlings in lined nests. The effect of *P. downsi* on nestling mass and tarsus length are consistent with other studies testing for effects of parasitic flies on nestling growth. In our study, nestlings in unlined nests weighed a mean of 13% less, and had tarsi that were a mean of 5% shorter than nestlings in lined nests. In comparison, nestling Blue tits (*Parus caeruleus*) and House wrens (*Troglodytes aedon*) parasitized by blowflies (*Protocalliphora*) weighed 3–6% less and had tarsi 0–2% shorter than unparasitized nestlings, prior to fledging [37], [38].

Our data show that experimentally reducing parasite abundance leads to a reduction in nestling body mass, tarsus length, and outermost primary feather length. Only the reduction in feather length was statistically significant; however, the fact that the effects on body mass and tarsus length were large in size, and in the same direction as the effect on feather size, suggests that *P. downsi* does, in fact, reduce nestling growth.

Our data showed no effect of parasitism on the bill sizes of nestlings, as estimated by a principal component analysis. However, the bill length, width and depth of *Geospiza* finches are known to increase more slowly than body mass, tarsus and wing chord [32]. Morphological traits such as flight feathers must grow quickly in order for nestlings to be capable of flying soon after they leave the nest. Similarly, nestlings with high body mass are more likely to survive after fledging than nestlings with low body mass [39]. *Geospiza* adults use their bills to crack seeds for food; however, seed cracking ability is not as important in young fledglings because adults continue feeding them after they leave nest [32].

Body size at fledging is known to predict post-fledging survival in birds [39], [40]. Therefore, it is likely that even a small effect of parasitism on nestling size prior to fledging will place birds at a significant disadvantage. Although we did not monitor post-fledging survival in our study, it is possible that fledglings from our unlined nests did not survive as well as the larger fledglings from lined nests. Thus, the impact of *P. downsi* on host reproductive success may have extended beyond the demonstrated impact on fledging success. Further study is needed to monitor post-fledging success in order to more fully understand long-term effects of *P. downsi* parasitism, in addition to the more immediate impact of the parasites on growth and fledging success.

While we did not test the effect of treatment on growth parameters repeatedly over the developmental period of the nestlings, the differences in growth were not apparent until nestlings were older in any case (Fig. 3A–C). The late appearance of growth differences between nestlings in lined and unlined nests may have been a byproduct of our method of parasite manipulation. *P. downsi* eggs and first instar larvae are often found in the nares (nostrils) of nestlings [19]. For this reason, the use of nylon liners would not necessarily affect the first instar stage of the parasite. It is possible that young nestlings in both lined and unlined nests experienced similar levels of first instar parasitism and, thus, similar effects on growth at an early age. In contrast, nest liners inhibited second and third instar larvae, which spend most of their time in the nest material. Thus, the impact on nestling size reported in our study may have been due primarily to second and third instar larvae.

*P. downsi* parasitism may affect nestlings through several non-mutually exclusive mechanisms. Blood-feeding parasites can lower hemoglobin concentrations in nestlings, causing anemia [41], [42]. Dudaniec *et al.* [21] found a negative correlation between *P. downsi* abundance and hemoglobin concentration in small ground finches (*G. fuliginosa*, Table 1). Fessl *et al.* [29] found that nestlings from parasitized nests tended to have lower hemoglobin concentrations than nestlings in unparasitized nests. Although we did not measure hemoglobin concentration in this study, our more recent work confirms that nestlings in parasitized nests have lower hematocrit (total red blood cell volume) than nestlings in unparasitized nests (Koop, unpublished data).

*P. downsi* may also affect nestling behavior and impede condition signaling to parents. Nestlings that are weakened by parasites may not have enough energy to beg for food [43]. Nestling begging intensity is correlated with the amount of food parents provide in other species of birds [44]. Even if nestlings are fed adequately, those in parasitized nests may suffer energetic costs that eventually lead to decreased survival. A recent study by O' Connor *et al.* [17] reported avoidance behaviors by nestling Darwin's finches toward *P. downsi* larvae in the nest. Larvae were most active at night; nestlings kept awake at night by feeding larvae presumably have less energy for growth. *P. downsi* larvae may also affect nestling growth indirectly by affecting parental behavior. Adult females irritated by feeding larvae, or by restless nestlings, may choose to stop brooding young, decrease feeding visits to the nest, or abandon the nest entirely. Further study is needed to investigate the proximal mechanisms underlying costs of *P. downsi* parasitism on fledging success.

Our study further demonstrates the devastating effect that *P. downsi* has on host fledging success. Only a single nest from the unlined treatment produced fledglings that were sighted after leaving the nest. A 42% experimental reduction in parasite abundance was sufficient to significantly increase the number of nests that fledged young. Thus, conservation efforts aimed at controlling *P. downsi* may be effective even if fly populations are simply reduced but not necessarily eliminated. Future monitoring is needed to determine whether the impact of *P. downsi* on nesting finches scales up to the level of populations and species [45], [46]. There is still much to learn about the ecology of *P. downsi* both in its native and introduced geographic ranges.
